# Dr. E. F. Semple

**Published:** 1886-04

**Authors:** 


### Dr. E. F. Semple.

Dr. E. F. Semple died suddenly at Dayton, O., February 27th, 1886, in the forty-first year of his age.

Dr. Semple's death was caused by heart disease, he was a member of Mad River Valley Dental Association, and has been in active practice since the late war. He was well liked by his professional brethren and the public in general, he leaves a wife and two children well provided for.

